# Bibliometric Analysis of the Epidemiological Research on Alzheimer’s Disease Treatment

**DOI:** 10.7759/cureus.87484

**Published:** 2025-07-07

**Authors:** Anayah Chowdhury, Garv Bhasin, Latha Ganti

**Affiliations:** 1 Research, Canterbury School, Fort Myers, USA; 2 Biology, Brown University, Providence, USA; 3 Medical Science, The Warren Alpert Medical School of Brown University, Providence, USA; 4 Emergency Medicine, Orlando College of Osteopathic Medicine, Winter Garden, USA; 5 Emergency Medicine & Neurology, University of Central Florida, Orlando, USA

**Keywords:** alzheimer's disease, bibliometric analysis, dementia research, epidemiology, global health

## Abstract

Alzheimer's disease presents a complex global health issue. It is characterized by a decline in cognitive function, starting with memory impairment, and extending to impact reasoning, language abilities, and spatial awareness. Despite decades of research, Alzheimer's disease remains a global challenge lacking long-term treatments. Institutions like the Karolinska Institutet, Columbia University, the University of California San Francisco (UCSF), and the University of Pittsburgh contribute significantly to Alzheimer's research, with a growth in publications in 2022 post-COVID-19. While current treatments offer symptomatic relief, there's a need for disease-modifying therapies targeting its mechanisms. This analysis aims to provide a comprehensive overview of the available research and medical literature on Alzheimer’s disease by employing bibliometric methods to identify publication trends, leading research institutions, and the evolving focus from symptomatic treatments to disease-modifying therapies. This paper seeks to analyze the research papers on Alzheimer’s disease and catalog the metadata associated with each paper.

## Introduction and background

Alzheimer's disease stands as a significant global health challenge, with dementia affecting an estimated 24 million individuals worldwide. This number is estimated to double every two decades until 2040 [1]. Alzheimer's is characterized by a progressive decline in cognitive activity and is the fifth-leading cause of death among Americans aged 65 years and older [2,3]. The disease begins with individuals having challenges in forming recent memories. As it progresses, its impact extends beyond memory, gradually affecting other cognitive functions such as reasoning, language, and spatial awareness [4]. This deterioration in the brain affects the individual’s capacity to perform everyday routines and tasks independently, eventually leading to complete dependency on caregivers for even the most basic activities of daily living, such as eating, bathing, and dressing [1]. Despite extensive research efforts, effective and long-term treatments remain elusive. This paper aims to identify research trends, institutional contributions, and gaps in the current understanding and treatment of Alzheimer's disease.

The human brain shrinks to a degree in normal aging, but does not lose neurons in large amounts. However, in Alzheimer’s, the damage is widespread, as many neurons stop functioning as they are meant to. These neurons lose connections with other neurons and eventually die [5]. The initial symptom in most Alzheimer's patients is memory loss, reflecting the early damage to neuronal connections within memory-related regions of the brain, such as the entorhinal cortex and hippocampus. As the disease progresses, it extends its impact to other areas of the cerebral cortex responsible for language, reasoning, and social abilities. Over time, widespread neuronal dysfunction and damage spread throughout the various regions of the brain [6]. Simple activities, such as managing finances, recognizing familiar faces, or following a conversation, become increasingly challenging. As the disease spreads to other parts of the brain, most individuals often experience personality changes, mood swings, and behavioral disturbances. Tragically, Alzheimer's disease is ultimately fatal.

The clinical criteria for the diagnosis of Alzheimer’s disease involve a gradual onset and worsening of memory and other cognitive functions over time. There are no motor, sensory, or coordination deficits early in the disease [7]. However, as it progresses, these deficits may become more apparent. Laboratory tests, including blood tests and brain imaging, are not definitive for diagnosing Alzheimer's disease but may be used to rule out other conditions that could mimic its symptoms, such as a subdural hematoma or frontotemporal dementia. Therefore, diagnosis often relies heavily on comprehensive neurological and neuropsychological tests, including detailed patient family history and cognitive testing. These evaluations help neurologists see a pattern of cognitive decline relating to Alzheimer's disease, confirming a more accurate diagnosis.

Alzheimer’s disease is one of the most complex neurodegenerative diseases [8]. It has no cure, and prevention remains the priority [9]. Some treatments can change the progression of the disease, and drug, or non-drug, options exist to help ease a patient’s symptoms. There are six approaches to the disease that might achieve genuine care. These are: 1) Therapies targeting reduced levels of TGF-β and Wnt/β-catenin, which decline as mild cognitive impairment (MCI) progresses to Alzheimer's, while also addressing impaired epithelial-to-mesenchymal transition (EMT) in the development of Alzheimer's; 2) Enhancing neuroprotective pathways that respond inadequately during the early stages of cognitive decline; 3) Stimulating compensatory brain mechanisms to counteract initial memory deficits; 4) Treatments targeting brain cells whose dysfunction leads to MCI and dementia; 5) Employing combination therapies even when a drug addresses a single cause like amyloid deposition; and 6) Increasing the chances of achieving a true cure by utilizing combinations of strategies selected from the aforementioned approaches [10]. 

## Review

Methods

Bibliometric analysis methods for the medical literature were employed [11]. The dataset collected was obtained using an advanced search on the Web of Science database for the keywords “Epidemiology” and “Alzheimer’s”, not case sensitive (TS = “Epidemiology” AND “Alzheimer’s”). A total of 2,055 articles were finally retrieved for analysis and exported as tab-delimited files with publication dates ranging from January 1986 to June 2024. Web of Science was chosen for its distinctive position as a well-known platform in global research, covering more than 1.9 billion cited references from 171 million records [12]. This database was particularly valuable for researching Alzheimer's disease due to its coverage of the published papers and ability to provide a clear view of research and advancements in this specific area of study. The Web of Science also contains a collection of bibliometric data, which can be imported into specialized software for analysis. For this study, VOSviewer 1.6.20 (Centre for Science and Technology Studies (CWTS), Leiden University, The Netherlands) was employed, a tool specifically designed for bibliometric analysis of articles. This paper analyzed which countries had the most publications on Alzheimer’s disease, the academic institutions with the most publications on the topic, the months each year that had the most publications or saw the greatest increase in publications, and the articles that had the most citations.

Results

Countries and Regions

**Figure 1:**
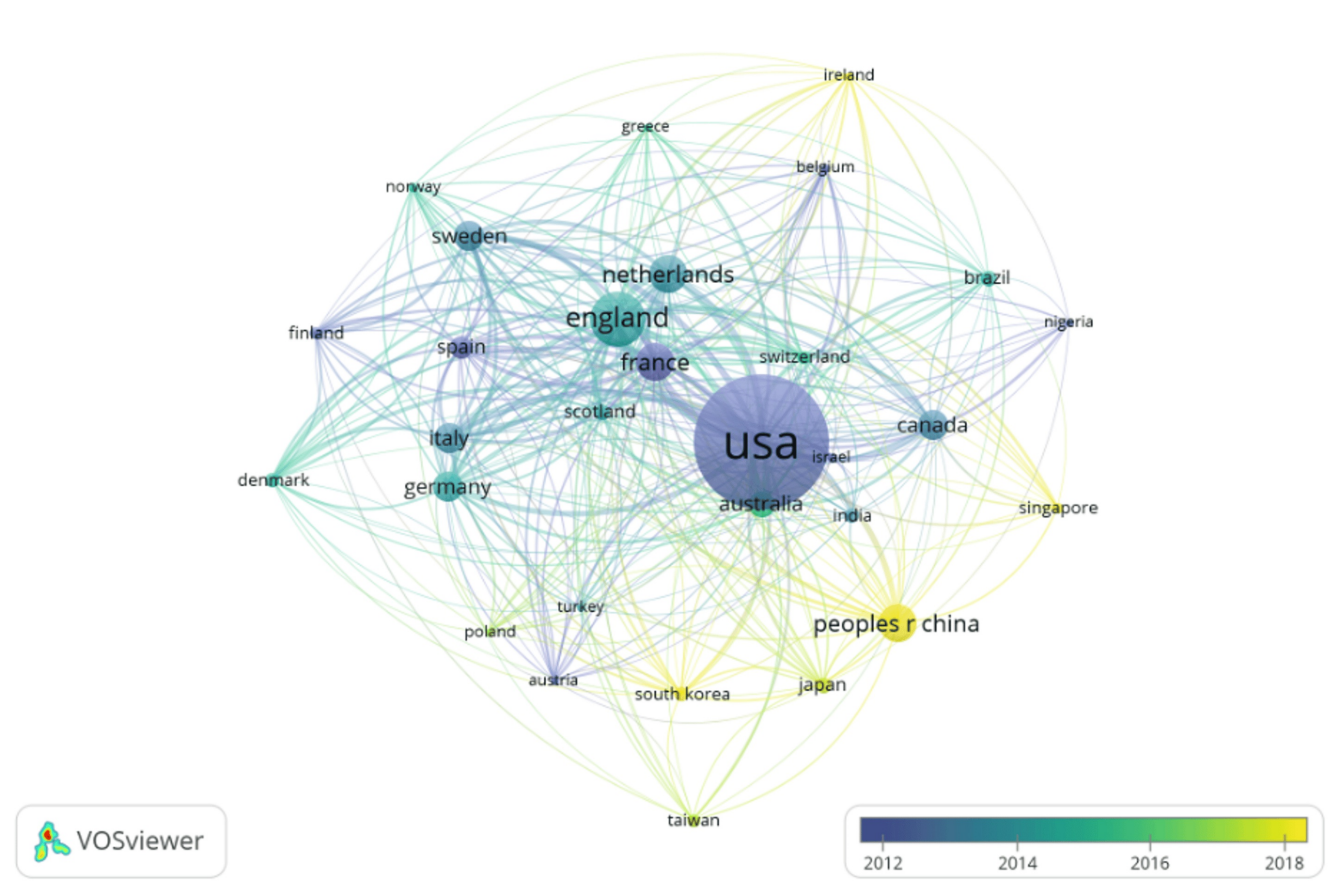
Geographical collaboration web diagram of publications

The country leading in total number of publications for this topic was the United States (US) at 40.1% (Figure 1).

**Figure 2:**
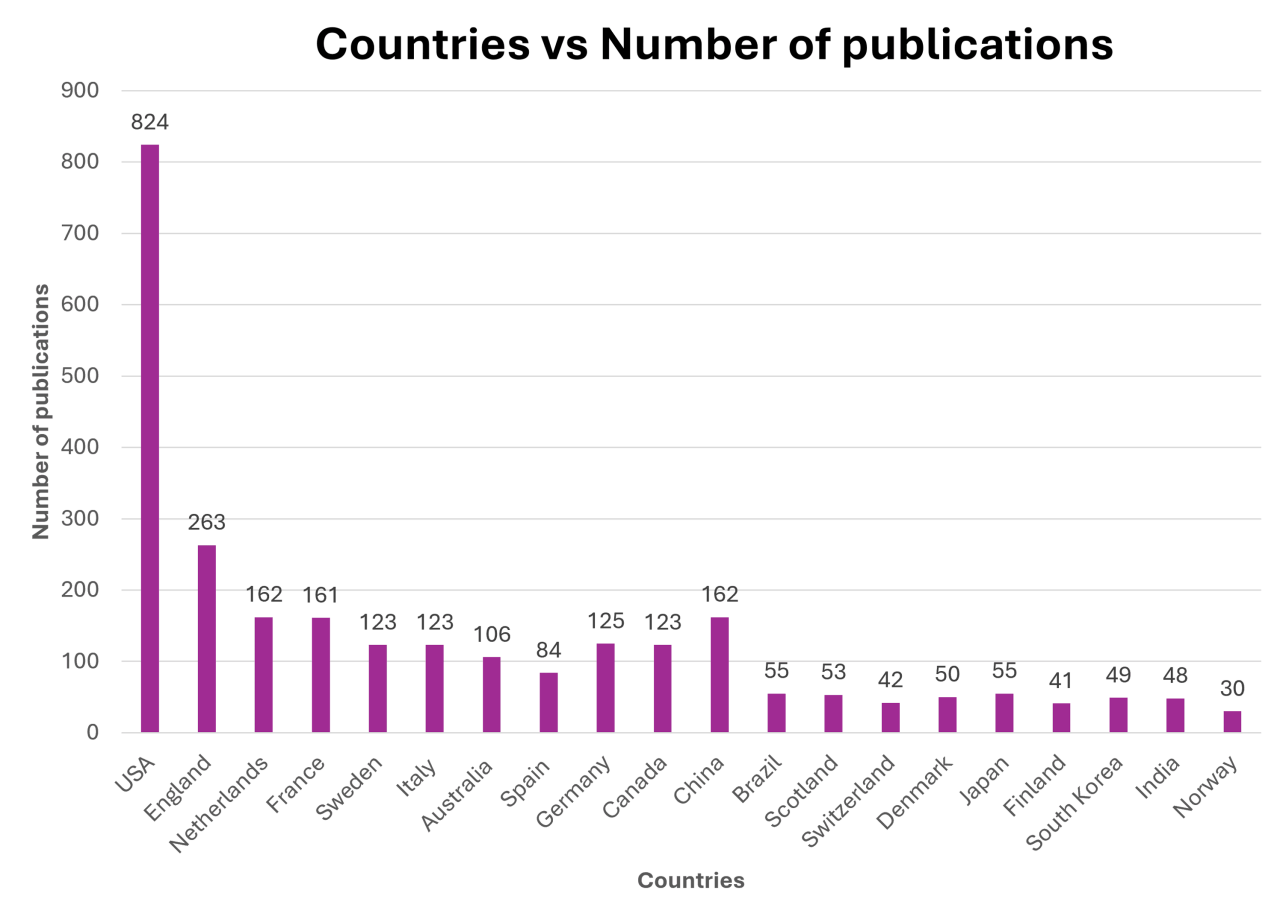
Publication bibliographic coupling by organizations

After the US, England and the Netherlands followed at 12.8% and 7.9%, respectively. France and Sweden were the fourth- and the fifth-largest contributors to Alzheimer’s publications with 7.8% and 6.0% of the total publications, respectively (Figure 2).

Although the US, England, and the Netherlands had the greatest number of publications, their papers tended to be older and published more in 2012. Some countries that had fewer but more recent publications included China, Singapore, Ireland, and South Korea.

Organizations and Institutions

**Figure 3:**
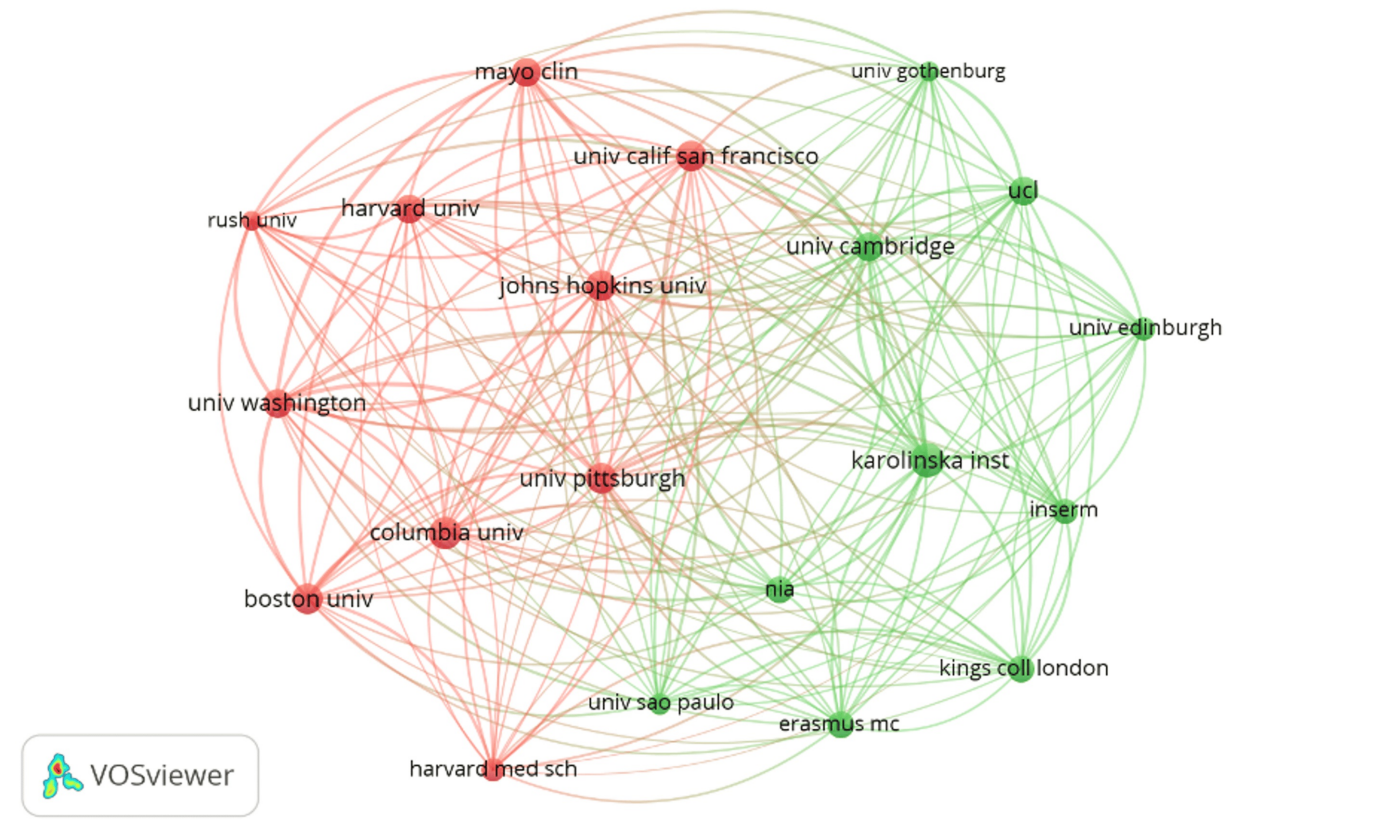
Bibliographic coupling of publications by organizations

The analysis of published articles revealed a notable concentration across several leading institutions. The number of published articles by these institutions was similar, resulting in a relatively less variation in the number of papers published by the first and the fifth institution on the list. This is unlike countries such as the US and France where there are significant differences between first and fourth leading countries in Alzheimer’s research. For instance, the Karolinska Institutet stood out as it published 62 papers on Alzheimer’s disease (Figure 3), indicating its significant contribution to the study of Alzheimer’s.

However, following it closely, was Columbia University, which ranked second with 58 publications. The University of California San Francisco (UCSF) and the University of Pittsburgh (Pitt) shared the third position, each contributing 54 publications. Boston University rounded out the top four with the publication of 49 papers. Other institutions reviewed in the analysis contributed 49 or fewer papers each.

Yearly Data and Analysis

**Figure 4:**
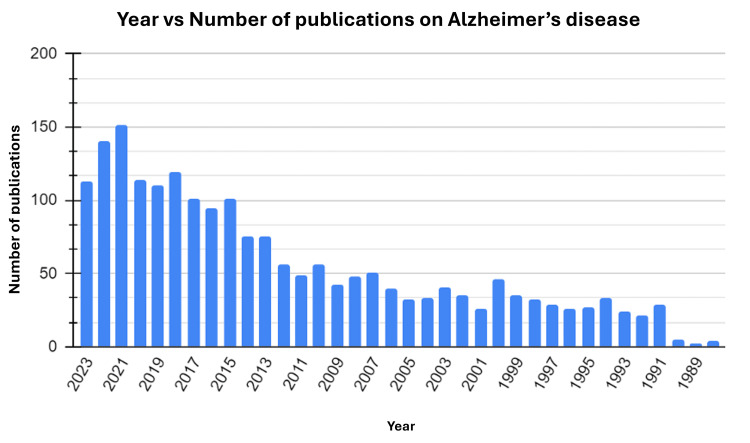
Bar chart of the number of the papers published each year

While indexing the articles by year, 1989, the second earliest year in the data collection, had the least number of publications (0.09%), while 2022 had the greatest number of publications (7.3%) (Figure 4).

The publications on Alzheimer’s disease saw the greatest increase in the years 2020 to 2021 (n=36). There was a significant drop from the year 2022 to 2023.

**Figure 5:**
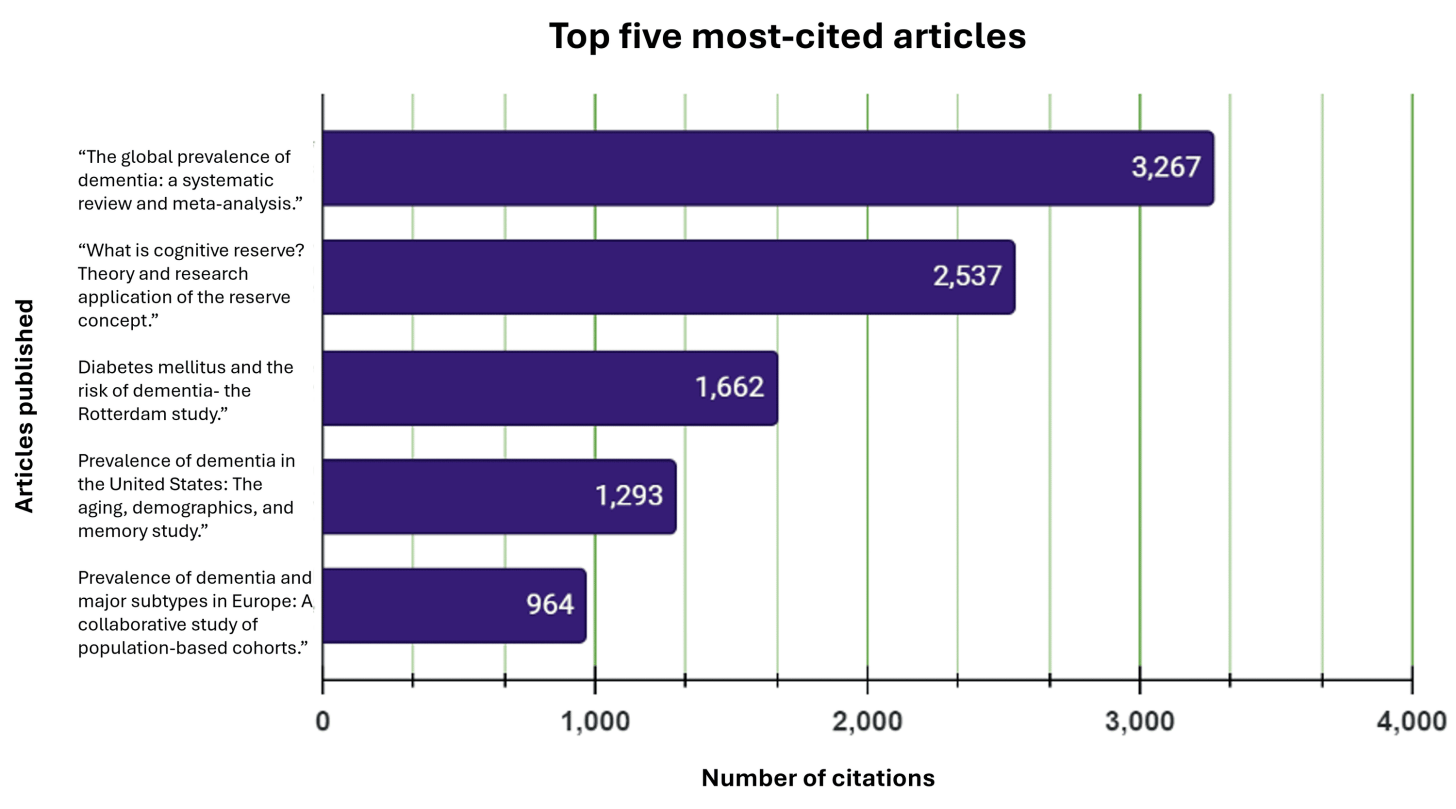
Stacked bar chart presenting the top five most-cited articles and the number of citations for each [13-17]

The top five most-cited articles were selected to explore their significance in Alzheimer’s disease. They were chosen based on their merit in contributing to the understanding and discussion of Alzheimer's in a medical and scholarly context. The most-cited article (3,267 citations) was “The global prevalence of dementia: A systematic review and metaanalysis” published in January 2013 (Figure 5) [13].

“What is cognitive reserve? Theory and research application of the reserve concept” [14] was the second most-cited article with 2,537 citations. It was followed by “Diabetes mellitus and the risk of dementia - The Rotterdam Study” [15], with 1,662 citations, “Prevalence of dementia in the United States: The aging, demographics, and memory study” [16] with 1,293 citations, and the “Prevalence of dementia and major subtypes in Europe: A collaborative study of population-based cohorts” [17] with 964 citations.

Discussion

Countries

In this section, it is important to note that adding up the total number of papers published by each country will exceed the total number cataloged by the Web of Science for the keywords put in. This is because the Web of Science logs certain papers as coming from multiple countries if the research was a joint effort. The leading country in publications was the US. The US is considered a nation that dedicates the greatest amount of funding to research compared to other countries [18]. Research on Alzheimer’s disease in the US is particularly prevalent, likely because aging is the biggest risk factor for dementia or Alzheimer’s disease. By 2050, 80 million Americans are projected to be 65 or older, up from 55 million in 2019 [19]. As the previous generations age, the number of cases of Alzheimer’s disease is expected to rise. The condition is also the sixth leading cause of death in the US, meaning more researchers are currently being hired to understand the disease and find new ways to treat it [20]. England has also historically had a large amount of research funding, and dementia is the leading cause of death amongst the English population. The Alzheimer’s Society in the United Kingdom (UK) invests 10 million euros annually in research projects and initiatives [21]. Supportive government policies and initiatives in England also promote Alzheimer’s research, encouraging institutions and organizations to contribute to this cause. The Netherlands followed England as the third most-published nation for Alzheimer's. The Netherlands is ranked seventh among the countries with the most cases of Alzheimer’s disease, with 33.78 cases for every 100,000 people [22]. Similar to the other Western countries on this list, the Netherlands faces challenges related to an aging population and healthcare needs associated with Alzheimer's disease. Therefore, the Dutch government invests significantly in research and development for this condition.

Organizations

The Karolinska Institutet is globally recognized for its research on medical and life sciences. The institution conducts research across various disciplines and even awards the Nobel Prize in physiology or medicine annually [23]. When analyzing the published articles, the Karolinska Institutet stood out as the top contributor to the topic. This is likely because it fosters collaborations with international universities, research institutions, and healthcare organizations worldwide. Columbia follows Karolinska as the second-largest contributor to the database. Columbia University hosts several research centers and institutes dedicated to neuroscience and neurodegenerative diseases. These centers conduct research aimed at understanding the mechanisms of Alzheimer's disease and developing new treatments. The university also offers educational programs that train the next generation of researchers and healthcare providers in Alzheimer's disease [24]. With this background, Columbia most likely published many papers on Alzheimer’s and dementia to educate others and foster collaboration among other universities. The UCSF and the Pitt are both the third most common contributors. The UCSF School of Medicine is one of the most research-intensive schools in the US and is renowned for its work in biomedical discovery [25]. Pitt also has a research program that focuses on neurodegenerative diseases like Alzheimer's, Parkinson's, and more [26]. UCSF and Pitt have strong ties to world-class medical centers and hospitals, which provide access to diverse populations of people and help students facilitate clinical trials and studies that are crucial to developing these papers on Alzheimer's.

Publication Years 

In 1989, one of the earliest years in the dataset, Alzheimer's disease research likely saw fewer publications due to the relatively early stage of scientific exploration. Given that Alzheimer's was only identified in 1906, research capabilities and methodologies, such as neuroimaging and genetic studies, were less advanced at that time compared to today. In contrast, 2022 experienced a significant increase in Alzheimer's publications. This increase can be attributed to the heightened awareness and research efforts that started after the COVID-19 pandemic. This intensified research across various medical fields, including in neurology and infectious diseases. The greatest growth in Alzheimer's publications occurred between 2020 and 2021. Initially, in 2020, the onset of the COVID-19 pandemic led to a redirection of research efforts away from Alzheimer's and towards COVID-19. However, by 2021, as COVID-19 vaccinations became available and research resumed, attention shifted back to neurodegenerative diseases like Alzheimer's, driving a notable increase in publications during that period. There was a significant drop from 2022 to 2023. After a peak of publications in 2022, attention may have shifted to new and emerging priorities in the field. It could also be due to more clinical studies occurring during this time, referencing the data and papers analyzed in 2022.

Limitations

This study's reliance on the Web of Science as its sole data source presents a significant limitation. Any study that is not present in that database is not included in our analysis, even if it falls within the search parameters. Despite Web of Science's extensive coverage, disparities with databases such as PubMed, especially concerning newer publications, are noteworthy. Web of Science requires a subscription for full access and is commonly accessed through institutional libraries or paid subscriptions. Additionally, this study focused exclusively on the keyword "Alzheimer’s," omitting other forms of dementia. Consequently, its capacity to offer a comprehensive portrayal of Alzheimer’s impact on medicine is somewhat constrained.

## Conclusions

Alzheimer's disease remains a pressing global health challenge. Despite decades of research, effective long-term treatments remain elusive. Insights from the bibliometric analysis revealed contributions from institutions like the Karolinska Institutet, Columbia University, UCSF, and Pitt. The analysis revealed fluctuations in publications with a significant increase observed in 2022, likely influenced by the heightened research efforts following the COVID-19 pandemic. However, a prominent decline in 2023 suggests shifting priorities within the field. While current treatments provide symptomatic relief, there is a critical need for disease-modifying therapies that can target disease mechanisms. Increased research investment enhances the likelihood of discovering more therapeutic targets, biomarkers for early diagnosis, and personalized treatment approaches for all patients with Alzheimer's disease.

## References

[1] (2024). 2024 Alzheimer's disease facts and figures. Alzheimers Dement.

[2] Mayeux R, Stern Y (2012). Epidemiology of Alzheimer disease. Cold Spring Harb Perspect Med.

[3] (2023). 2023 Alzheimer's disease facts and figures. Alzheimers Dement.

[4] Corey-Bloom J (2002). The ABC of Alzheimer's disease: cognitive changes and their management in Alzheimer's disease and related dementias. Int Psychogeriatr.

[5] Larson EB, Kukull WA, Katzman RL (1992). Cognitive impairment: dementia and Alzheimer's disease. Annu Rev Public Health.

[6] (2025). What happens to the brain in Alzheimer’s disease?. https://www.nia.nih.gov/health/alzheimers-causes-and-risk-factors/what-happens-brain-alzheimers-disease.

[7] McKhann G, Drachman D, Folstein M, Katzman R, Price D, Stadlan EM (1984). Clinical diagnosis of Alzheimer's disease: report of the NINCDS-ADRDA Work Group under the auspices of Department of Health and Human Services Task Force on Alzheimer's Disease. Neurology.

[8] Breijyeh Z, Karaman R (2020). Comprehensive review on Alzheimer's disease: causes and treatment. Molecules.

[9] Passeri E, Elkhoury K, Morsink M (2022). Alzheimer's disease: treatment strategies and their limitations. Int J Mol Sci.

[10] Fessel J (2023). The several ways to authentically cure Alzheimer's dementia. Ageing Res Rev.

[11] Ganti L, Persaud NA, Stead TS (2025). Bibliometric analysis methods for the medical literature. Acad Med Surg.

[12] Clarivate - Web of Science Group. (2022, August 31 (2025). Scientific and academic research. Clarivate. https://clarivate.com/academia-government/scientific-and-academic-research/.

[13] Prince M, Bryce R, Albanese E, Wimo A, Ribeiro W, Ferri CP (2013). The global prevalence of dementia: a systematic review and metaanalysis. Alzheimers Dement.

[14] Stern Y (2002). What is cognitive reserve? Theory and research application of the reserve concept. J Int Neuropsychol Soc.

[15] Ott A, Stolk RP, van Harskamp F, Pols HA, Hofman A, Breteler MM (1999). Diabetes mellitus and the risk of dementia: the Rotterdam Study. Neurology.

[16] Plassman BL, Langa KM, Fisher GG (2007). Prevalence of dementia in the United States: the aging, demographics, and memory study. Neuroepidemiology.

[17] Lobo A, Launer LJ, Fratiglioni L (2000). Prevalence of dementia and major subtypes in Europe: a collaborative study of population-based cohorts. Neurology.

[18] Hale Hale, A. B. a (2025). The State of U.S. Science and Engineering 2022. National Science Foundation. Science and Engineering.

[19] (2025). United States. The Aging Readiness & Competitiveness Report 5.0. https://www.aarpinternational.org/initiatives/aging-readiness-competitiveness-arc/united-states.

[20] (2025). Alzheimer's disease facts and figures. Alzheimer's Association. https://www.alz.org/alzheimers-dementia/facts-figures.

[21] (2025). What research is Alzheimer’s Society funding?. https://www.alzheimers.org.uk/research/our-research/what-research-alzheimers-society-funding.

[22] (2025). Alzheimer's rates by country 2025. https://worldpopulationreview.com/country-rankings/alzheimers-rates-by-country.

[23] (2025). The Nobel prize in physiology or medicine. Karolinska Institutet. https://ki.se/en/about-ki/prizes-and-ceremonies/prizes-and-awards/the-nobel-prize-in-physiology-or-medicine.

[24] Alzheimer’s Disease Research Center (ADRC). (2024, January 25 (2025). Alzheimer’s Disease Research Center (ADRC). Columbia University. https://www.neurology.columbia.edu/research/research-centers-and-programs/alzheimers-disease-research-center-adrc.

[25] Research Research (2025). Research. University of California San Francisco School of Medicine. https://medschool.ucsf.edu/research.

[26] (2025). About PIND. Pittsburgh Institute For Neurodegenerative Diseases. https://www.pind.pitt.edu/about.

